# Transferring an Adult-Plant Stripe-Rust Resistance Gene *Yr7VS* from Chromosome 7V of *Dasypyrum villosum* (L.) to Bread Wheat

**DOI:** 10.3390/plants13131875

**Published:** 2024-07-07

**Authors:** Fu Hou, Yinyu Jin, Jin Hu, Lingna Kong, Xiaoxue Liu, Liping Xing, Aizhong Cao, Ruiqi Zhang

**Affiliations:** 1 State Key Laboratory of Crop Genetics & Germplasm Enhancement and Application/JCIC-MCP, College of Agronomy, Nanjing Agricultural University, Nanjing 210095, China; 2 Huaiyin Institute of Agricultural Sciences of Xuhuai Area in Jiangsu, Huaian 223001, China; 3 Zhongshan Biological Breeding Laboratory, No.50 Zhongling Street, Nanjing 210014, China

**Keywords:** *D. villosum*, T7DL·7V#6S translocation line, stripe rust, APR gene *Yr7VS*

## Abstract

Stripe rust (*Puccinia striiformis* West. f.sp. *tritici*, *Pst*) is a destructive disease that seriously threatens wheat production globally. Exploring novel resistance genes for use in wheat breeding is an urgent need, as continuous *Pst* evolution frequently leads to a breakdown of host resistance. Here, we identified a set of wheat–*Dasypyrum villosum* 01I139 (V#6) disomic introgression lines for the purpose of determining their responses to a mixture of *Pst* isolates CYR32, CYR33 and CYR34 at both seedling and adult-plant stages. The results showed that all introgression lines exhibited high susceptibility at the seedling stage, with infection-type (IT) scores in the range of 6–8, whereas, for chromosomes 5V#6 and 7V#6, disomic addition lines NAU5V#6-1 and NAU7V#6-1 displayed high resistance at the adult-plant stage, indicating that adult-plant resistance (APR) genes were located on them. Further, in order to transfer the stripe-rust resistance on chromosome 7V#6, four new wheat–*D. villosum* introgression lines were identified, by the use of molecular cytogenetic approaches, from the self-pollinated seeds of 7D and 7V#6, in double monosomic line NAU7V#6-2. Among them, NAU7V#6-3 and NAU7V#6-4 were t7V#6L and t7V#6S monosomic addition lines, and NAU7V#6-5 and NAU7V#6-6 were homozygous T7DS·7V#6L and T7DL·7V#6S whole-arm translocation lines. Stripe-rust tests and genetic analyses of chromosome 7V#6 introgression lines revealed a dominant APR gene designated as *Yr7VS* on the chromosome arm 7V#6S. Comparison with the homozygous T7DL·7V#6S translocation line and the recurrent parent NAU0686 showed no significant differences in yield-related traits. Thus, T7DL·7V#6S whole-arm translocation with the APR gene *Yr7VS* provided a valuable germplasm for breeding for resistance.

## 1. Introduction

Stripe rust, caused by *Puccinia striiformis* West. f.sp. *tritici*, is one of the most damaging diseases affecting wheat (*Triticum aestivum* L.) production worldwide. Average yield losses per year caused by stripe rust range from 5–10%, but yield losses of more than 70% can occur in highly susceptible varieties [1]. The development and deployment of cultivars with host resistance is the most economical and environmentally friendly approach to the reduction of wheat-yield losses caused by stripe rusts. However, the resistance genes employed could turn out to be ineffective, as virulence in the pathogen population could be selected or the pathogen might rapidly evolve when a certain resistance gene is extensively used over large areas [2]. Therefore, ongoing efforts to exploit novel and effective sources of resistance are essential to counter the continuous evolution of stripe-rust pathogens.

Plants have developed various types of resistance to pathogens through their long-term coevolution [3]. In the case of stripe rust, race-specific all-stage resistance (ASR) genes, as well as race-nonspecific adult-plant resistance (APR) genes, have been employed in wheat breeding [4]. Race-specific ASR genes, such as *Yr10* and *Yr28*, typically encode nucleotide-binding leucine-rich repeat (NLR) receptors that recognize specific pathogen avirulence (Avr) proteins and trigger a hypersensitive reaction [5,6]. The majority of these genes are non-durable as to their effectiveness, due to the evolution of new forms of virulence in the pathogen. In contrast, race-nonspecific APR genes, such as *Yr18* and *Yr46*, encoding an ATP binding cassette (ABC) transporter and a hexose transporter involved in sugar uptake, respectively, confer durable resistance [7,8]. Although more than 86 stripe-rust resistance genes have been explored in wheat and its wild relatives (https://wheat.pw.usda.gov/GG3/wgc) (The access date is 1 May 2024), most of these genes belong to the race-specific ASR category. Therefore, the exploration of novel APR genes contributes to breeding durable resistance to wheat stripe-rust.

Numerous wheat disease-resistance genes have been introgressed from the plant’s wild relatives, enriching the genetic diversity of modern wheat [9]. For example, wheat stripe-rust resistance genes *Yr9* and *Yr83* originated from *Secale* cereal [10,11], *Yr50* and *YrT14* were introgressed from *Thinopyrum intermedium* [12,13] and *Yr69* was introgressed from *Thinopyrum ponticum* [14]. Of these, *Dasypyrum villosum* (2n = 2x = 14, VV), an annual wild relative of wheat, has been used as a source for wheat improvement for decades [15]. Six *D. villosum* accessions (V#1 to V#6) were used to develop the amphiploids and alien addition/substitution lines [15,16]. Utilizing these intermediate materials, dozens of wheat–*D. villosum* translocation lines have been developed through centric breakage–fusion between homoeologous chromosomes or through physical irradiation [15]. Notably, seven powdery mildew genes, *Pm21*, *Pm55*, *Pm62*, *Pm67*, *Pm5V*, *Pm3VS* and *Pm4VL*, have been characterized and introgressed into bread wheat through the development of Robertsonian translocations [17,18,19,20,21,22,23]. Among them, the genes *Pm21*, *Pm55* and *Pm5V*, typically encoding NLR receptors, have been cloned, and *Pm55* and *Pm5V* were determined to be allelic and renamed as *Pm55a* and *Pm55b*, respectively [3,18]. In addition, the stripe-rust reactions of 115 *D. villosum* accessions have been tested, and 41 of them (35.65%) were resistant to different *Pst* isolates [24]. However, only a stripe rust ASR gene *YrCD-3* on chromosome 3V#3 and an APR gene *Yr5V* on chromosome arm 5V#5S have been introgressed from *D. villosum* into bread wheat [19,25]. Recently, a complete array of new introgression lines involving chromosomes 1V#6 to 7V#6 of *D. villosum* accession 01I139 in the cv. NAU0686 genetic background have been developed [23], but their responses to stripe rust are unclear.

Here, we evaluated the stripe-rust responses for the chromosomes 1V#6 to 7V#6 as to disomic introgression lines, at both the seedling and adult-plant stages. The objectives of this work were to identify strip rust APR genes on the *D. villosum* chromosomes, and introgress them from the V genome into the wheat genomes through the breakage–fusion mechanism. Our results will provide a potential resource for wheat breeding with reference to resistance to stripe rust.

## 2. Results

### 2.1. Stripe-Rust Responses of 1V#6 to 7V#6 Disomic Lines

**Figure 1:**
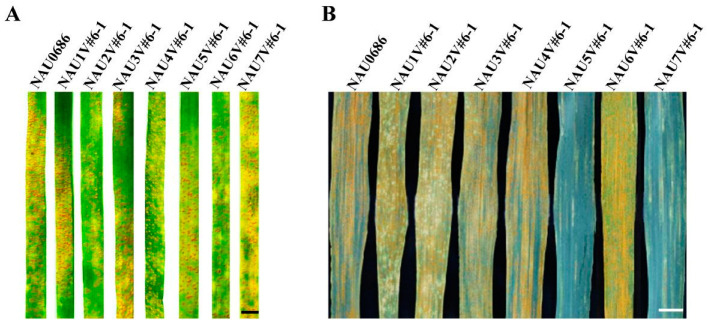
Stripe-rust reactions of a set of wheat–*D. villosum* disomic introgression lines involving chromosomes 1V#6 to 7V#6. (**A**) In the seedling (three-leaf) stage, recurrent parent NAU0686 and seven disomic introgression lines were highly susceptible (IT 6-8) to the mixed isolates of CYR32, CYR33 and CYR34. Bar: 0.5 cm. (**B**) Lines NAU0686, NAU1V#6-1, NAU2V#6-1, NAU3V#6-1, NAU4V#6-1 and NAU6V#6-1 were susceptible to stripe rust at the adult-plant stage, while lines NAU5V#6-1 and NAU7V#6-1 were resistant. Bar: 0.5 cm.

To explore the stripe-rust resistance genes introduced from *D. villosum* accession 01I139, seven disomic lines of chromosomes 1V#6 to 7V#6 were assessed for their reactions to stripe rust at both the seedling and adult-plant stages. The results showed that the seven introgression lines, as well as their background parent NAU0686, were all susceptible to the mixed *Pst* strains (CYR32, CYR33 and CYR34) with an IT score of 6–8 at the seedling stage (Figure 1A). However, chromosomes 5V#6 and 7V#6, disomic addition lines NAU5V#6-1 and NAU7V#6-1, exhibited high resistance to the mixed *Pst* strains at the adult-plant stage, with DS values of 10 and 20, respectively (Figure 1B). These findings suggest that the genes conferring resistance to stripe rust at the adult-plant stage are likely located on chromosomes 5V#6 and 7V#6, respectively. Previously, a stripe rust APR gene, *Yr5V*, was mapped to the short chromosome arm 5V#5S. This APR gene identified on chromosome 5V#6 may be allelic to it. However, no genes conferring resistance to stripe rust on chromosome 7V of *D. villosum* have been reported.

### 2.2. Development of a Double Monosomic Line of Chromosomes 7D and 7V#6

**Figure 2:**
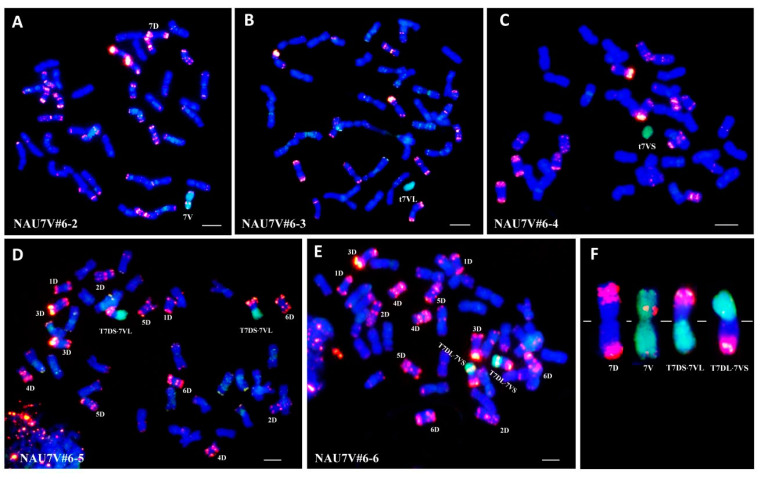
Identification of chromosome 7V#6 introgression lines through genomic in situ hybridization (GISH) and fluorescent in situ hybridization (FISH). *D. villosum* 01I139 genomic DNA labeled with fluorescein-12-dUTP (*green*) as probes was used for GISH. The D-genome-specific probe for FISH was Oligo-pAs1-2,5, labeled with TAM (*red*). Wheat chromosomes were counterstained with DAPI (*blue*). (**A**) GISH/FISH patterns of NAU7V#6-2 (2n = 42), showing double monosomic aspects for chromosomes 7D and 7V#6. (**B**) GISH/FISH patterns of NAU7V#6-3 (2n = 43), t7V#6L monosomic addition line. (**C**) GISH/FISH patterns of NAU7V#6-4 (2n = 43), t7V#6S monosomic addition line. (**D**) GISH/FISH patterns of NAU7V#6-5 (2n = 42), homozygous T7DS·7V#6L translocation line. (**E**) GISH/FISH patterns of NAU7V#6-6 (2n = 42), homozygous T7DL·7V#6S translocation line. (**F**) Comparison of chromosomes 7D, 7V#6, T7DS·7V#6L and T7DL·7V#6S. Bars: 10 μm.

**Figure 3:**
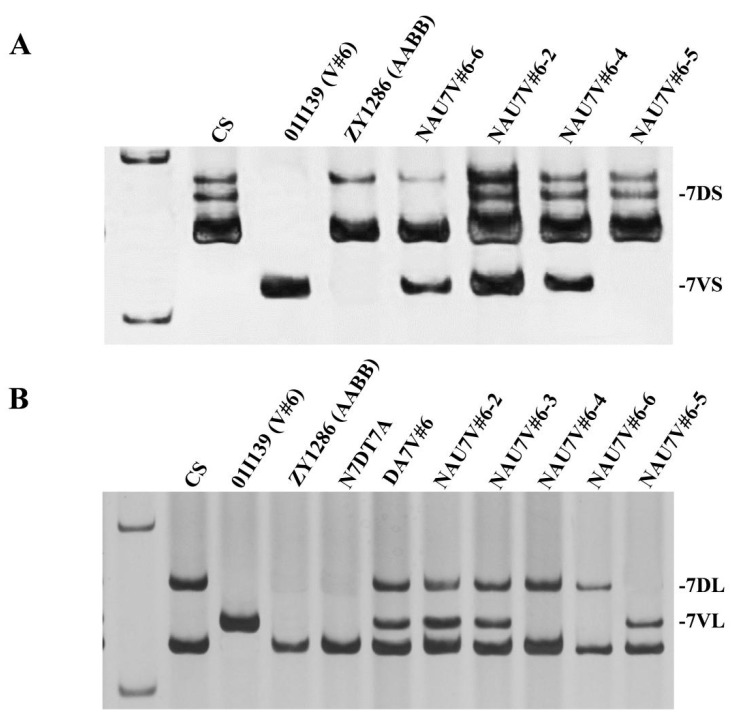
PCR amplification patterns of wheat–*D. villosum* introgression lines using specific molecular markers. (**A**) PCR amplification patterns, using 7VS-specific marker *CINAU7VS-166*. (**B**) PCR amplification patterns, using 7VL-specific marker *CINAU7VL-67*. Marker: DL2000.

To produce a line with a double monosomic condition involving chromosomes 7D and 7V#6, disomic addition line NAU7V#6-1 (2n = 44) was used as the female parent in a cross with the NT line N7DT7A (2n = 42), derived from CS. In the F_2_ progeny, a plant designated as NAU7V#6-2 was identified by GISH/FISH analysis; this plant had a chromosome number of 2n = 42, containing a single chromosome 7D and a single chromosome 7V#6 (Figure 2A). This line was further confirmed using 7V/7D co-dominant molecular markers *CINAU7VL-67* and *CINAU7VS-166*, which indicated the presence of specific bands for both chromosomes, namely, 7D and 7V#6 (Figure 3A,B). Therefore, NAU7V#6-2 was a double monosomic line and the Robertsonian translocations might be present in its self-pollinated seeds, and potentially formed through the breakage–fusion mechanism between chromosomes 7D and 7V#6.

### 2.3. Development of Chromosome 7V#6 Alterations

To identify the Robertsonian translocations, a total of 285 plants obtained from the self-pollinated seeds of chromosomes 7D and 7V#6, double monosomic line NAU7V#6-2, were screened using 7VS- and 7VL-specific molecular markers *CINAU7VL-67* and *CINAU7VS-166*. Of these, 16 plants exhibited polymorphic bands only for the 7VS-specific marker, while 17 plants displayed polymorphic bands only for the 7VL-specific marker. Subsequent GISH/FISH analysis revealed that 13 of 16 7VS-plants contained a 7V#6S telosome; the remaining three plants were heterozygous with a normal 7D chromosome and a T7DL·7V#6S whole-arm translocated chromosome. Additionally, 15 of 17 7VL-plants had a 7V#6L telosome, and the remaining two plants contained a T7DS·7V#6L whole-arm translocated chromosome. Consequently, genetically fixed lines derived from these plants included t7V#6L monosomic addition line NAU7V#6-3 (Figure 2B), t7V#6S monosomic addition line NAU7V#6-4 (Figure 2C), homozygous T7DS·7V#6L translocation line NAU7V#6-5 (Figure 2D,F) and homozygous T7DL·7V#6S translocation line NAU7V#6-6 (Figure 2E,F).

### 2.4. Chromosome Location of the Stripe-Rust Resistance Conferred by 7V#6

**Figure 4:**
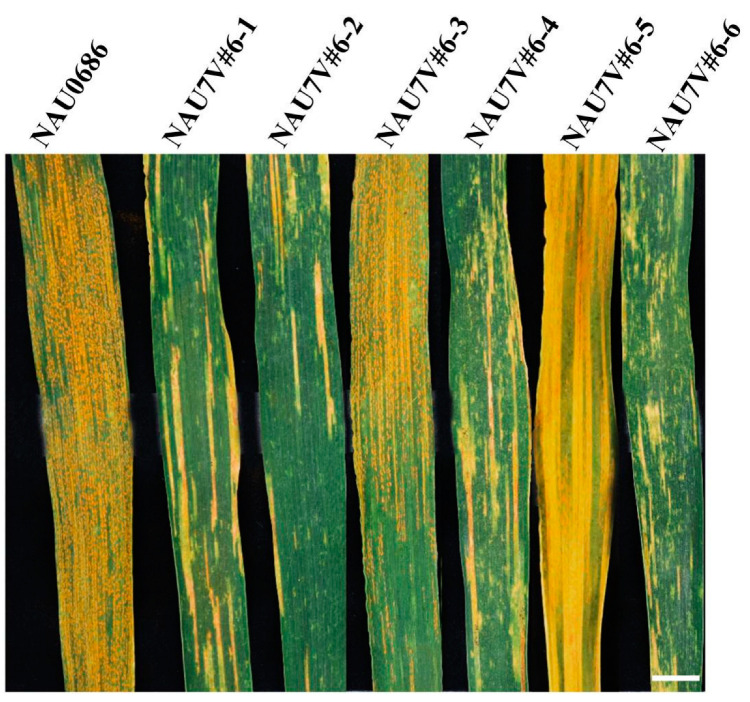
Stripe-rust reactions of chromosome 7V#6 introgression lines at the adult-plant stage. Lines NAU7V#6-1, NAU7V#6-2, NAU7V#6-4 and NAU7V#6-6 exhibited high resistance to a mixture of *Pst* isolates CYR32, CYR33 and CYR34 at the adult-plant stage, whereas NAU7V#6-3 and NAU7V#6-5 were susceptible. Bar: 0.5 cm.

In order to localize the resistance on chromosome 7V#6, the four introgression lines of NAU7V#6-3, NAU7V#6-4, NAU7V#6-5 and NAU7V#6-6 were inoculated using the mixed isolates of races CYR32, CYR33 and CYR34. All lines were identified as being susceptible to stripe rust at the seedling stage in the greenhouse (IT 7-8). When testing them at the adult-plant stage in the field nursery, the 7V#6L-related lines NAU7V#6-3 and NAU7V#6-5 were still susceptible, while 7V#6S-related lines NAU7V#6-4 and NAU7V#6-6 exhibited high resistance, suggesting that the stripe rust APR gene could be located on the chromosome arm 7V#6S (Figure 4).

**Figure 5:**
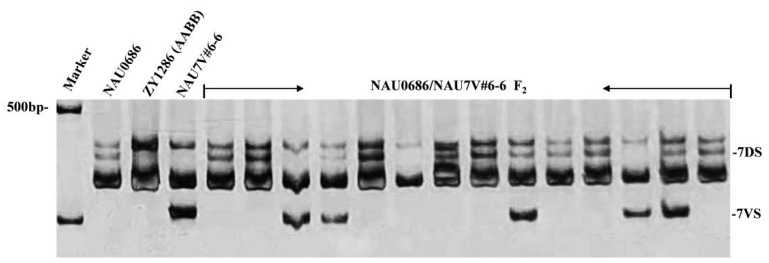
PCR amplification patterns of 7VS/7DS co-dominant marker *CINAU7VS-166* in NAU0686/NAU7V#6-6 F_2_ individuals.

To further confirm whether the resistance was linked to the alien fragment of T7DL·7V#6S, a cross was conducted between NAU0686 and NAU7V#6-6. All F_1_ plants (n = 20) showed adult-plant resistance, with an average DS value of 23.5 (10–30). Subsequently, the F_2_ plants were identified using the 7VS-specific marker (Figure 5). Among the 302 plants identified, 69 individuals lacked alien chromatin, 161 were heterozygous, and 72 were disomic for a T7DL·7V#6S recombinant chromosome pair, suggesting normal gametic transmission of the translocated chromosome relative to its intact chromosome 7D (χ^2^_1:2:1_ = 1.3, *p* > 0.05) (Table 1). These plants were then tested for their reaction to the stripe rust. All of the plants with the T7DL·7V#6S translocated chromosome exhibited high resistance at the adult-plant stage, whereas the 69 plants lacking T7DL·7V#6S were all susceptible, displaying an average DS value of 76.5 (Table 1).

**Figure 6:**
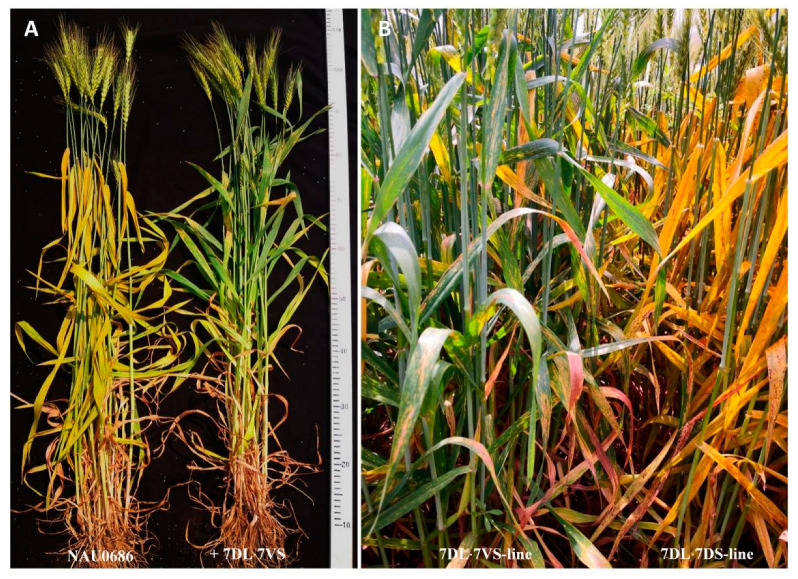
The morphologies of adult plants with or without T7DL·7V#6S translocation. (**A**) Plant morphology of T7DL·7V#6S translocation line and its recurrent parent NAU0686, showing the similar developmental stages and plant height shared between them. (**B**) The 7DL·7DS and 7DL·7V#6S NILs in the NAU0686 background, showing enhanced resistance to stripe rust caused by the T7DL·7V#6S introgression.

Furthermore, the backcrossing lines with or without the T7DL·7V#6S translocated chromosome were identified from BC_3_F_2_ individuals of NAU7V#6-6/3*NAU0686. Ten lines (BC_3_F_3_) with homozygous T7DL·7V#6S-translocated chromosomes were compared as to their responses to stripe rust with another ten lines, each with a pair of chromosomes 7D. The results confirmed that all the T7DL·7V#6S-lines exhibited high resistance to stripe rust at the adult-plant stage (DS 20-30), whereas all the 7DL·7DS-lines were highly susceptible, with DS scores ranging from 60 to 100 (Figure 6A,B), confirming that the stripe-rust resistance originated from *D. villosum*. Taken together, we concluded that a dominant APR gene is located on chromosome arm 7V#6S; this gene was temporary designated as *Yr7VS.*

### 2.5. Evaluation of Major Agronomic Traits

**Figure 7:**
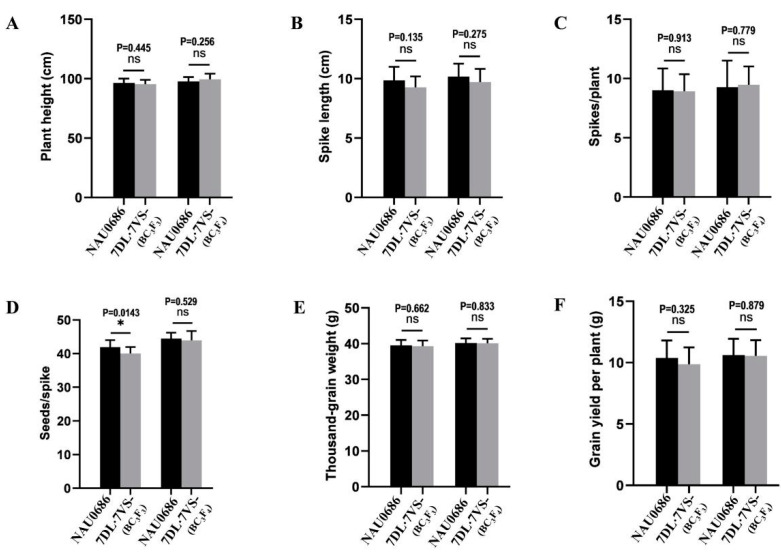
Comparative analysis of agronomic traits between T7DL·7V#6S translocation lines and recurrent parent NAU0686. (**A**–**F**) The differences of the yield-related traits in plant height (**A**), spike length (**B**), spikes/plant (**C**), grains/spike (**D**), thousand-grain weight (**E**) and grain yield per plant (**F**), showing that the T7DL·7V#6S translocation would not have significant negative impacts on agronomic traits. Values are the mean ± SD (two-sided t-test; * *p* < 0.1; ns not significant; n = 45).

The T7DL·7V#6S translocation line possesses the stripe rust APR gene, suggesting that it could be an excellent resource for wheat improvement. However, it remains unknown whether it has any linkage drag making it undesirable relative to major agronomic traits. To investigate whether the T7DL·7V#6S translocated chromosome has any negative effect on the agronomic traits of bread wheat, we compared the yield-related traits between the BC_3_F_3_ and BC_3_F_4_ homozygous T7DL·7V#6S lines and their recurrent parent NAU0686 (Figure 7). The results showed that the T7DL·7V#6S-lines exhibited normal fertility and developmental stages, similar to the recurrent parent NAU0686, under field conditions (Figure 6A). Statistical analyses revealed no significant differences between the BC_3_F_3_ homozygous T7DL·7V#6S line and NAU0686 in terms of plant height, spike length, spikes per plant, thousand-grain weight, or grain yield per plant, but the grains per spike of the T7DL·7V#6S line was a little lower than that of NAU0686. In comparison, the BC_3_F_4_ homozygous T7DL·7V#6S line showed no significant differences in any of the tested agronomic traits. Accordingly, the T7DL·7V#6S translocation would not have significant negative impacts on agronomic traits, making it a valuable resource for wheat improvement.

## 3. Discussion

Exploiting genes from wild relatives is an important means used to broaden wheat’s genetic diversity [9]. *D. villosum* is an annual outcrossing diploid grass species which displays a high level of genetic differentiation among natural populations. Although the hybrids between tetraploid wheat and *D. villosum* are easy to produce, only a fraction of the alien chromatin is currently available as introgressions into bread wheat [15,16]. Recently, a complete set of new wheat–*D. villosum* V#6 disomic introgression lines were developed in CINAU, including four disomic substitution lines (2n = 42) containing, respectively, chromosomes 1V#6, 2V#6, 3V#6 and 6V#6, in addition to four disomic addition lines (2n = 44) containing, respectively, chromosomes 4V#6, 5V#6, 6V#6 and 7V#6 [23]. These lines have been evaluated as to their responses to powdery mildew at both the seedling and adult-plant stages [17,23]. In the present study, we further evaluated their responses to stripe rust, and revealed a novel stripe rust APR gene *Yr7VS* on the short arm of chromosome 7V#6. The identification of a compensating T7DL·7V#6S whole-arm translocation line with high levels of resistance to stripe rust at the adult-plant stage may provide a useful resource for wheat improvement.

Robertsonian whole-arm translocations (RobTs), which facilitate alien gene mapping to chromosome arms, are an initial material basis for small-fragment chromosome engineering; in addition, they can be used to evaluate the agronomic performance of the alien introgressed genes [19]. To develop RobTs translocations, the centric breakage–fusion behavior of univalents can be exploited [26]. In a double monosomic plant consisting of an alien chromosome and a homoeologous wheat chromosome, both monosomes remain univalent at meiotic metaphase I and have a tendency to break at the centromeres. This can result in the formation of RobTs through fusion of the broken arms [27,28]. By producing plants which were double-monosomic for chromosomes 7D of wheat and 7V#6 of *D. villosum*, we targeted the formation of RobTs between these two chromosomes. Seven RobTs were identified among the 280 progenies double-monosomic for the screened chromosomes 7D and 7V#6, corresponding to a frequency of 2.5%. The same approach was used to produce RobTs involving wheat chromosomes 1D to 7D and *D. villosum* chromosomes 1V#5 to 7V#5 [19].

Studies have shown that introgressed alien genes may be orthologous to resistance genes in wheat; examples include rye-derived *Pm8* and wheat *Pm3* [29], as well as *D. villosum Pm21* and wheat *Pm12* [30]. To date, several cataloged stripe-rust resistance genes in wheat and its relatives have been located on homoeologous group 7 chromosome short arms. Among them, *Yr18* on 7DS confers a pleiotropic resistance effect [31]. *Yr2* on chromosome 7B is a recessive gene [32], while *Yr6* on chromosome arm 7BS in cv. Fielder is a seedling resistance gene [33]. However, in the present study, a stripe rust APR gene, *Yr7VS*, was confirmed as being located on chromosome arm 7V#6S; this could be distinct from previously characterized genes on the homoeologous group 7 chromosome short arms. Although genetic analysis in F_2_ and F_2:3_ progenies of cross NAU7V#6-6/NAU0686 exhibited a dominant gene conferring stripe-rust resistance at the adult-plant stage, and the gene was located on chromosome arm 7V#6S (Table 1), the number of introgressed alien resistance genes on this chromosome arm is unclear, as no recombinations have been identified between 7VS and 7DS. Thus, isolation and molecular-mechanism analysis of the gene *Yr7V* is needed, and this work will be performed in the next research effort.

Genome sequencing has revealed complex translocation events involving 4VL-5VL-7VS in *D. villosum*, which is similar to the 4AL-5AL-7BS translocation in wheat [34,35]. Notably, the size of the segment translocated from 7VS to 4VL was found to be larger than that of the fragment translocated from 7BS to 4AL in wheat [35]. In the current study, two Robertsonian whole-arm translocations, T7DS·7V#6L and T7DL·7V#6S, were developed through the fusion of the broken arms of chromosomes 7D and 7V#6. These lines might potentially have an impact on the genetic diversity and agronomic traits of wheat. However, yield-related-traits evaluation showed that T7DL·7V#6S translocations have no significant negative effects on major yield-related traits, which suggests that compensatory effects from genes on 7DS, missing in the T7DL·7V#6S translocation, could be provided by homologs on the homoeologous chromosome arms 7AS or 7BS. Overall, the development of the T7DL·7V#6S translocation line NAU7V#6-6 represents a valuable novel genetic resource for attempts to enhance stripe-rust resistance in wheat breeding. The stripe rust APR gene *Yr7VS* can be combined with other major or minor resistance genes to achieve low rust severity and durability [36]. Molecular markers for cloned ASR genes, such as *Yr10*, and APR genes, such as *Yr36*, are available and can be used for gene stacking [37,38]. We are presently transferring the T7DL·7V#6S translocation to commercial cultivars, which will allow us to evaluate its effects in other genetic backgrounds.

## 4. Materials and Methods

### 4.1. Plant Materials

The materials used in this study are listed in Table 2. Among them, *D. villosum* accession 01I139 (#6) was the donor of the alien introgression lines. Durum wheat ZY1286 (2n = 4x = 28, AABB) was, firstly, crossed with 01I139 to develop the amphiploid line AABBVV-3 [17]. Bread wheat NAU0686, with a high susceptibility to stripe rust, was used as a recurrent parent. A set of wheat–*D. villosum* disomic chromosome 1V#6 to 7V#6 lines was developed from the progenies of AABBVV-3/4*NAU0686 [23]. To develop translocation lines, the chromosome 7V#6 disomic addition line was crossed as a female with the Chinese Spring (CS)-derived nullisomic/tetrasomic (NT) line N7DT7A provided by the Wheat Genetics and Genomics Resource Centre, Kansas State University, Manhattan, KS, USA. Their progenies were screened for double monosomic plants with chromosomes 7D of wheat and 7V#6 of *D. villosum* accession 01I139. The new introgression lines were identified from the self-pollinated progenies of the 7D/7V double monosomic plant by molecular cytogenetic approaches. All of the genetic resources were maintained at the Cytogenetics Institute, Nanjing Agricultural University (CINAU).

### 4.2. Cytogenetic Analysis

The chromosomes of wheat and *D. villosum* were detected by genomic in situ hybridization (GISH) and fluorescence in situ hybridization (FISH) techniques. Root-tip cells from the germinating seedlings of the introgression lines were used to prepare the mitotic metaphase chromosomes [39]. *D. villosum* accession 01I139 genomic DNA was labeled with fluorescein-12-dUTP (green), used as a probe for GISH detection [40]. The special DNA repeat sequence Oligo-pAs1-2,5, labeled with 6-carboxytetramethylrhodamine (TAM) and synthesized by Shanghai Sangon Biotech Co., Ltd. (Shanghai, China), was used as probe for FISH detection, which produces red signals and preferentially paints tandem repeats on the wheat D genome. All of the chromosomes on the slides were counterstained with 4,6-diamidino-2-phenylindole (DAPI) (Invitrogen Life Science, Carlsbad, CA, USA) with a blue signal. Cytological images were captured using an Olympus BX60 microscope (Olympus Co., Tokyo) equipped with a SPOT Cooled Color Digital Camera (Diagnostic Instruments, Sterling Heights, MI, USA).

### 4.3. Molecular Marker Analysis

Leaf samples were collected at the 3-leaf stage, and genomic DNA was extracted using the cetyl trimethyl ammonium bromide (CTAB) method. The 7VL-specific molecular markers *CINAU7VL-67* (F: GACTCTTCAGCCAGTTACTTCA; R: CTGCAGCGCCTCCTTTCC) and 7VS-specific molecular markers *CINAU7VS-166* (F: CACAGTCAACAAGGAGGTCC; R: CCCAGTGCTGCTTGATGAAC) developed by Zhang et al. [41] were used to screen the progenies of NAU7V#6-1/N7DT7A for selection of the lines carrying chromosome arms 7VS or 7VL. PCR amplification was conducted using an iCycler thermalcycler (Bio-RAD Laboratories, Emeryville, CA, USA) in a 10 μL reaction volume. The reaction mixture contained 40 ng of genomic DNA, 2.0 μmol/L of each primer, 2.5 mmol/L of each dNTP and 0.2 U of Taq DNA polymerase, following the protocol described by Zhang et al. [19]. Subsequently, the PCR amplification products were combined with 2.0 μL of loading buffer (comprising 10 mM EDTA, 98% formamide, 0.25% bromophenol blue and 0.25% xylene cyanol) and subjected to analysis in 8% non-denaturing polyacrylamide gels. The D2000 Plus DNA Ladder (GenStar, Beijing, China) was used for the DNA marker in non-denaturing polyacrylamide gel. The resulting band patterns were visualized using silver staining.

### 4.4. Stripe-Rust Evaluation

The stripe-rust responses of wheat–*D. villosum* introgression lines and their recurrent parent NAU0686 were assessed at both seedling and adult-plant stages. The mixed *Pst* strains, including CYR32, CYR33 and CYR34 isolates, from types currently prevailing in China, were used to infect the plants of the test lines and the spreader [42]. The seedling tests were performed in a greenhouse environment with a temperature of 16 °C and a light intensity of 150–170 µmol m^−2^ s^−1^ for 16 h, followed by 8 h at 10 °C in darkness. Ten seeds from each of the lines were grown in rectangular trays, with each tray consisting of 32 cells (6.5 × 6.5 × 9.0 cm). The susceptible check NAU0686 was planted with three cells randomly selected from among the trays. Seedling infection-type (IT) data were recorded 14 days post-inoculation, utilizing a modified 0–9 scale, when the spores were fully developed on the first affected leaf of the susceptible cultivar NAU0686 [43].

The same set of lines was tested at the adult-plant stage against the mixture of stripe-rust pathotypes. All of the materials were planted in the field nurseries at the Baima Experiment Station of Nanjing Agricultural University. Twenty seeds from each line were sown in individual 1.5 m rows with 25 cm spacing between them. The plants were rated for stripe-rust infection at the shooting stage. NAU0686 samples were used as the susceptible control. Adult-plant reactions were assessed using a 0–100 scale, based on the severity of the disease levels on the flag leaves of the susceptible check when they had reached maximum severity [44].

### 4.5. Evaluation of Agronomic Traits

Homozygous T7DL·7V#6S translocation lines were identified from the BC_3_F_2_ progenies of the NAU7V#6-6/3*NAU0686. Yield trials of BC_3_F_3_ and BC_3_F_4_ lines and the recurrent parent NAU0686 were performed in the experimental station of Jiangsu Academy of Agricultural Sciences, Nanjing, China. Each line was arranged in a randomized plot design, with rows (1.5 m × 0.2 m/row) with three replications. All of the plants were treated with fungicides and pesticides to manage diseases and insects. At the physiological maturity stage, fifteen plants located in the middle of the internal two rows of T7DL·7V#6S translocation lines and NAU0686 were randomly chosen for the analysis of various yield-related traits, including plant height (PH), spike length (SL), spike number per plant, grains per spike, and thousand-grain weight (TKW). The mean values and standard errors of the treatments were determined using Microsoft Excel. Differences in agronomic traits between the lines were evaluated using Tukey’s post hoc test (SPSS 26.0, Chicago, USA) at a significance level of *p* = 0.05.

## 5. Conclusions

In this study, a complete set of wheat–*Dasypyrum villosum* 01I139 (V#6) disomic introgression lines were evaluated with reference to their responses to stripe rust at both seedling and adult-plant stages. By using a combination of GISH/FISH and molecular markers, wheat–*D. villosum* homozygous T7DS·7V#6L translocation line NAU7V#6-5, and T7DL·7V#6S translocation line NAU7V#6-6 were identified. A stripe rust APR gene, *Yr7VS*, located on the chromosome arm 7VS#6, was confirmed and introgressed into the wheat. The T7DL·7V#6S translocation showed no obvious negative effect on yield-related traits; this research provides a new germplasm which can be used in breeding for resistance.

## Figures and Tables

**Table 1:** Adult-plant stage segregation for reaction to stripe rust in progeny of cross NAU7V#6-6/NAU0686.

Chromosome Status	(7VS/7VS)	(7VS/7DS)	(7DS/7DS)	Expected Ratio	*χ* ^2^
Number of F_2_ individuals	72	161	69	1:2:1	1.3
Strip-rust severity (%) of individual F_2_ plants	20 (10–30)	28.5 (20–50)	76.5 (60–100)	-	-
Response of F_2:3_ lines	Homozygous resistant	Segregating	Homozygous susceptible	-	

*χ*^2^ value for significance at *p* = 0.05 is 5.99.

**Table 2:** The genetic stocks developed and used in this study.

Line	Chromosome Structure	Description
NAU0686	AABBDD (2n = 42)	High-yield wheat cultivar highly susceptible to stripe rust; used as the recurrent parent for all alien introgression lines
N7DT7A	Null 7D (2n = 42)	Nulli-tetrasomic line of Chinese Spring
01I139	V#6V#6 (2n = 14)	Donor *Dasypyrum villosum* collected from Greece
ZY1286	AABB (2n = 28)	Spring durum wheat used to cross with *D. villosum* 01I139
NAU7V#6-1	DA7V#6 (2n = 44)	NAU0686, with an added pair of chromosome 7V#6 of 01I139
NAU7V#6-2	MA7V#6 (2n = 42)	7D and 7V#6, double monosomic line
NAU7V#6-3	t7V#6L (2n = 43)	7V#6L telosomic addition line
NAU7V#6-4	t7V#6L (2n = 43)	7V#6S telosomic addition line
NAU7V#6-5	T7DS·7V#6L (2n = 42)	Compensation translocation line in which chromosome arm 7V#6L is substituted for 7DL
NAU7V#6-6	T7DL·7V#6S (2n = 42)	Compensation translocation line in which chromosome arm 7V#6S is substituted for 7DS

## Data Availability

The original contributions presented in the present study are included in the article. The plant materials and datasets generated and analyzed during the present study are available from the corresponding authors upon reasonable request.
